# CpG Frequency in the 5′ Third of the *env* Gene Determines Sensitivity of Primary HIV-1 Strains to the Zinc-Finger Antiviral Protein

**DOI:** 10.1128/mBio.02903-19

**Published:** 2020-01-14

**Authors:** Dorota Kmiec, Rayhane Nchioua, Scott Sherrill-Mix, Christina M. Stürzel, Elena Heusinger, Elisabeth Braun, Marcos V. P. Gondim, Dominik Hotter, Konstantin M. J. Sparrer, Beatrice H. Hahn, Daniel Sauter, Frank Kirchhoff

**Affiliations:** a Institute of Molecular Virology, Ulm University Medical Center, Ulm, Germany; b Department of Medicine, University of Pennsylvania, Philadelphia, Pennsylvania, USA; c Department of Microbiology, University of Pennsylvania, Philadelphia, Pennsylvania, USA; University of Washington

**Keywords:** CpG dinucleotides, envelope gene, human immunodeficiency virus, restriction factors, zinc-finger antiviral protein

## Abstract

CpG dinucleotide suppression has been reported to allow HIV-1 to evade inhibition by the zinc-finger antiviral protein (ZAP). Here, we show that primate lentiviruses display marked differences in CpG frequencies across their genome, ranging from 0.44% in simian immunodeficiency virus SIVwrc from Western red colobus to 2.3% in SIVmon infecting mona monkeys. Moreover, functional analyses of a large panel of human and simian immunodeficiency viruses revealed that the magnitude of CpG suppression does not correlate with their susceptibility to ZAP. However, we found that the number of CpG dinucleotides within a region of ∼700 bases at the 5′ end of the *env* gene determines ZAP sensitivity of primary HIV-1 strains but not of HIV-2. Increased numbers of CpGs in this region were associated with reduced *env* mRNA expression and viral protein production. ZAP sensitivity profiles of chimeric simian-human immunodeficiency viruses (SHIVs) expressing different HIV-1 *env* genes were highly similar to those of the corresponding HIV-1 strains. The frequency of CpGs in the identified *env* region correlated with differences in clinical progression rates. Thus, the CpG frequency in a specific part of *env*, rather than the overall genomic CpG content, governs the susceptibility of HIV-1 to ZAP and might affect viral pathogenicity *in vivo*.

## Abstract (precis)

Evasion of the zinc-finger antiviral protein (ZAP) may drive CpG dinucleotide suppression in HIV-1 and many other viral pathogens but the viral determinants of ZAP sensitivity are poorly defined. Here, we examined CpG suppression and ZAP sensitivity in a large number of primate lentiviruses and demonstrate that their genomic frequency of CpGs varies substantially and does not correlate with ZAP sensitivity. We further show that the number of CpG residues in a defined region at the 5′ end of the *env* gene together with structural features plays a key role in HIV-1 susceptibility to ZAP and correlates with differences in clinical progression rates in HIV-1-infected individuals. Our identification of a specific part of *env* as a major determinant of HIV-1 susceptibility to ZAP restriction provides a basis for future studies of the underlying inhibitory mechanisms and their potential relevance in the pathogenesis of AIDS.

## INTRODUCTION

It has long been known that many RNA viruses mimic the suppression of CpG dinucleotides of their vertebrate hosts and that an increased CpG abundance in the genomes of influenza A virus, picornaviruses, and retroviruses is detrimental to their replication (1–5). Recent data suggest that the zinc-finger antiviral protein (ZAP, also known as ARTD13, PARP13, and ZC3HAV1) may be one of the driving forces behind the suppression of CpG dinucleotides in vertebrate RNA viruses (6). It has been shown that ZAP binds to regions in HIV-1 mRNAs with high CpG content to target them for degradation, thereby reducing viral protein expression and replication (6). In addition, experimental introduction of CpGs into the HIV-1 genome significantly increased its susceptibility to ZAP (6). Indeed, evasion of ZAP restriction may drive CpG suppression in many viral pathogens since ZAP exerts broad antiviral activity, inhibiting retro-, alpha-, filo-, hepadna-, and flaviviruses as well as retroelements (6–13).

The conclusion that CpG suppression allows HIV-1 to escape the antiviral effects of ZAP is based on derivatives of a single, cell-culture-adapted, subtype B strain of the pandemic M (major) group of HIV-1 (6). However, there are four different groups of HIV-1 and nine groups of HIV-2. Furthermore, related primate lentiviruses have been detected in more than 40 African primate species (14–16). These simian immunodeficiency viruses (SIVs) are genetically highly diverse and have been infecting their natural primate hosts for millions of years (14–16). HIV-1 group M strains, which are responsible for the global AIDS pandemic, resulted from a single zoonotic transmission of SIVcpz infecting chimpanzees about a hundred years ago (14). Chimpanzee viruses also gave rise to the rare HIV-1 group N strains and to SIVgor found in gorillas, the source of epidemic group O and extremely rare group P HIV-1 strains. In addition, SIVsmm naturally infecting sooty mangabeys crossed the species barrier to humans on at least nine occasions, although only two of these transmissions resulted in significant spread in the human population, giving rise to HIV-2 groups A and B (14).

Here, we examined CpG suppression and sensitivity to ZAP inhibition of a broad variety of HIV and SIV strains. We found that the magnitude of CpG suppression varies substantially between primate lentiviral lineages and that the genomic frequency of CpGs does not correlate with lentiviral sensitivity to ZAP inhibition. Moreover, we identified significant differences in CpG content in lentiviruses before and after cross-species transmission from monkeys to great apes or humans. Functional and mutational analyses of infectious molecular clones of primary HIV-1 strains revealed that ZAP sensitivity is determined by the number of CpGs in the 5′ third of the *env* gene rather than the overall CpG frequency in the viral genome. However, this association was observed only for HIV-1 and not for other primate lentiviruses, suggesting complex means of ZAP interaction with viral RNAs and/or mechanisms of evasion or counteraction. Together with structural RNA features, the frequency of CpGs in the identified *env* region also affected HIV-1 susceptibility to endogenous ZAP expression and correlated with differences in clinical progression rates, suggesting a potential role in HIV-1 pathogenicity *in vivo*.

## RESULTS

### Primate lentiviruses differ in the magnitude of CpG suppression.

**FIG 1:**
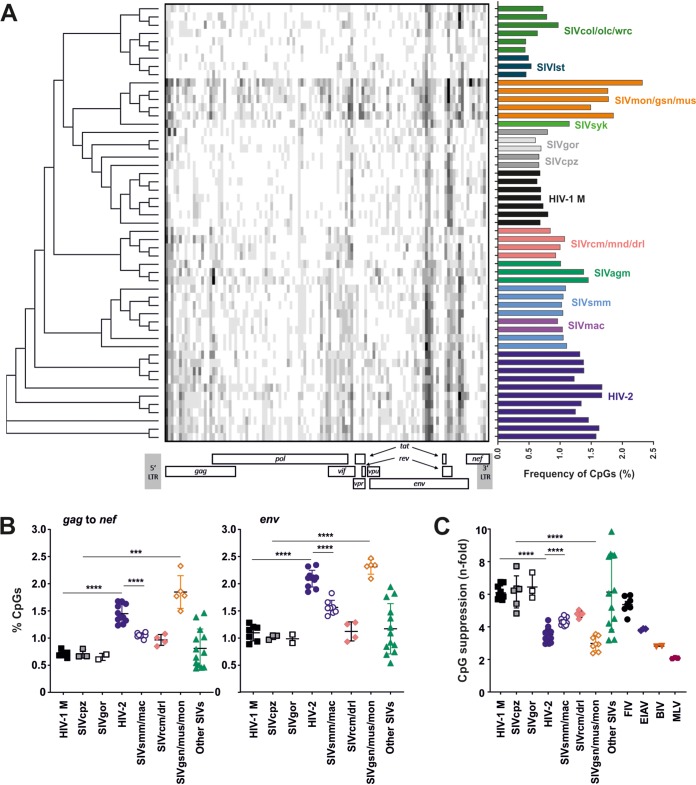
Comparison of CpG dinucleotide distribution and frequency in primate lentiviral genomes. (A) (Left) Cladogram showing the relationship between representative primate lentiviruses based on gene coding nucleotide sequence, followed by CpG frequency heatmap showing the number of CpG dinucleotides (white = 0 CpGs, black = 9 CpGs) per 100 bp in aligned lentiviral genomes. Relative position of CpGs is shown in reference to the HIV-1 genome (below). (Right) Frequency of CpG dinucleotides in the lentiviral genomes (number of CpGs divided by the length of the genomic region). LTR, long terminal repeat. (B) Comparison of the frequency of CpG dinucleotides in the coding genome region from *gag* to *nef* (left) or the *env* gene (right). Lentiviral sequences shown in panel A were grouped according to their phylogenetic relationship, and statistically significant differences were calculated using Student’s *t* test. ***, *P* < 0.001; ****, *P* < 0.0001. (C) Reduction (*n*-fold) of the frequency of CpG dinucleotides in retroviral genomes compared to the frequency predicted based on the viral genomic GC content.

To determine the level of CpG dinucleotide suppression in different primate lentivirus lineages, we examined 53 near-full-length genomes of HIV-1 and HIV-2, their SIVcpz and SIVgor or SIVsmm precursors, respectively, and a variety of other SIVs infecting African monkey species (Fig. 1A; see also Table S1 in the supplemental material). This analysis revealed substantial lineage-specific differences in the frequency and distribution of CpG residues, ranging from 0.4% in SIVwrc infecting Western red colobus monkeys to 2.3% in SIVmon infecting mona monkeys (Fig. 1A). Humans, apes, and monkeys show relatively little variation in the mean GC content (17) and mean CpG frequencies (2.3% to 2.7%) of their mRNA (18) (data not shown). Thus, various CpG frequencies in primate lentiviruses cannot be explained by differences in the CpG levels in their respective hosts. HIV-1 and its ape precursors, SIVcpz and SIVgor, all showed frequencies of CpG dinucleotides ranging from 0.6% to 0.8% (Fig. 1B, left), which is ∼6-fold lower than predicted from their mean GC content (Fig. 1C). In contrast, the SIVgsn/mus/mon group exhibited ∼3-fold-lower CpG frequencies than expected from their genomic GC content (Fig. 1C). It is thought that the *env* gene of SIVcpz originated from a precursor of today’s SIVgsn/mus/mon lineage (16, 19). Thus, our results suggest a significant drop in CpG frequency in this genomic region after transmission of SIV from monkeys to hominoids (Fig. 1B). In contrast, HIV-2 strains showed significantly higher CpG content and lower CpG suppression than their SIVsmm precursors as well as HIV-1 strains (Fig. 1B and C). Thus, our analyses indicate that the viral CpG content dropped after transmission from monkeys to apes but increased after transmission of SIVsmm to humans.

10.1128/mBio.02903-19.8 TABLE S1 CpG frequencies in primate lentiviral genomes. Download Table S1, DOCX file, 0.02 MB. Copyright © 2020 Kmiec et al. 2020 Kmiec et al. This content is distributed under the terms of the Creative Commons Attribution 4.0 International license.

### CpG content does not correlate with primate lentiviral susceptibility to ZAP inhibition.

**FIG 2:**
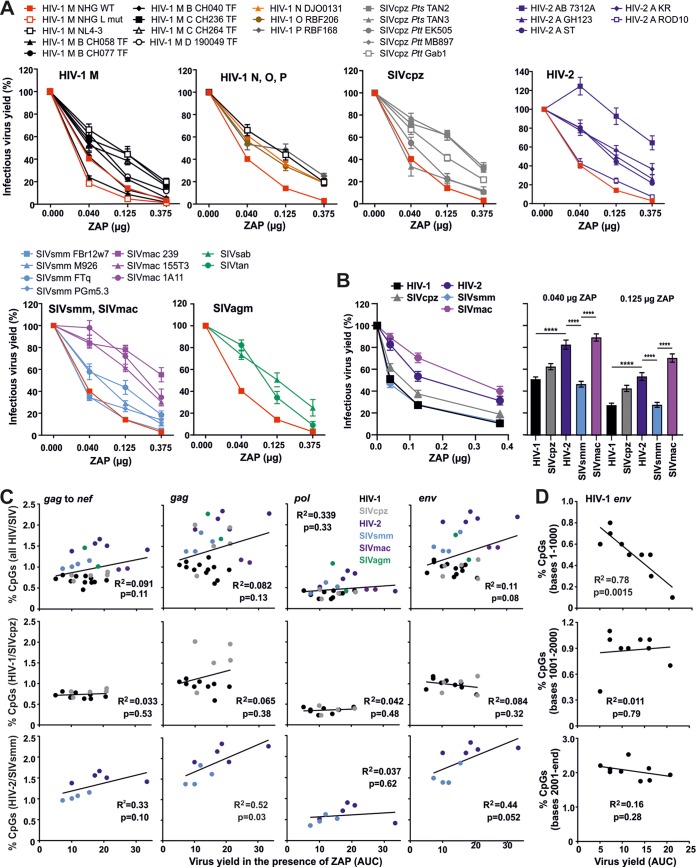
Primate lentiviral sensitivity to human ZAP. (A) Proviral constructs of the indicated infectious molecular clones of HIV-1, HIV-2, or SIV and increasing amounts of a plasmid expressing the N-terminally HA-tagged long isoform of human ZAP were cotransfected into HEK293T ZAP KO cells. Infectious virus yield was measured using the TZM-bl reporter cell infectivity assay. For each proviral construct, values were normalized to the infectious virus yield obtained in the absence of ZAP (100%). Shown is the mean from 5 independent experiments measured in triplicates ± SEM. (B) Group comparison of lentiviral sensitivity to ZAP. Values shown represent the average infectious virus yield relative to the absence of ZAP (100%) for the indicated groups of primate lentiviruses. Bar diagram shows the infectious virus yield obtained for all IMCs from the indicated groups at low and intermediate levels of ZAP expression. ****, *P* < 0.0001, calculated using Student’s *t* test. (C) Correlation between the frequency of CpGs in viral genomes (*gag* to *nef*) and *gag*, *pol*, or *env* genes in the genomes of primate lentiviruses analyzed and the corresponding virus yield in the presence of ZAP. Color coding: HIV-1, black; SIVcpz, gray; HIV-2, dark blue; SIVsmm, light blue; SIVmac, purple; SIVagm, green. AUC (area under the curve) was calculated from inhibition curves as shown in panel A. (D) Correlation between the number of CpG dinucleotides in the indicated regions of the HIV-1 *env* gene and the infectious virus yield in the presence of ZAP. Symbols in all panels represent the average value obtained for one IMC from the indicated groups in five experiments. Correlations were calculated with the linear regression module of Prism software.

Takata and colleagues (6) increased the CpG content of HIV-1 and showed that this conferred higher susceptibility to ZAP. To determine the sensitivity of primary HIV-1 strains and other primate lentiviruses to ZAP, we analyzed a panel of 31 infectious molecular clones (IMCs) of HIV and SIV (Fig. 2A). Our collection included six primary IMCs of HIV-1 group M as well as one clone (each) of HIV-1 groups O, N, and P; five strains of SIVcpz from eastern and central chimpanzees; five clones of HIV-2 and four of its SIVsmm counterpart; three strains of SIVmac and two SIVs infecting tantalus or sabaeus monkeys. To test the susceptibility of these IMCs to ZAP, we measured infectious virus yield from ZAP knockout (KO) HEK293T cells following cotransfection of the proviral constructs with different doses of a ZAP expression (or empty) vector. Control experiments showed that ZAP overexpression did not cause cytotoxic effects (Fig. S1A) and that all proviral constructs produced significant quantities of infectious virus (Fig. S1B). All HIV-1 strains were inhibited by ZAP in a dose-dependent manner (Fig. 2A, upper left). In agreement with published data (6), the HIV-1 NHG L mutant containing artificially high levels of CpGs was more sensitive to ZAP than the wild-type (WT) NHG virus. The susceptibility of group M HIV-1 strains to ZAP varied. While the CH058 transmitted-founder (TF) HIV-1 IMC was almost as sensitive as the HIV-1 NHG L mutant, most TF HIV-1 strains were less susceptible to ZAP (Fig. 2A). Group O, N, and P HIV-1, as well as SIVcpz strains, did not differ significantly in their ZAP sensitivity from group M HIV-1 IMCs (Fig. 2A, top, and Fig. 2B, right).

10.1128/mBio.02903-19.1 FIG S1 (A) Viability of HEK293T ZAP KO cells following transfection with the indicated amounts of ZAP expression or empty control vector determined by CellTiter-Glo assay. (B) HEK293T ZAP KO cells were transfected with the indicated HIV and SIV proviral constructs and empty pCG IRES BFP vector. Quantity of infectious HIV-1 in the culture supernatants obtained 2 days later was determined by infecting TZM-bl indicator cells. Shown are average values ± SEM (*n* = 5) for infectious virion yield derived from triplicate infections. Download FIG S1, TIF file, 2.2 MB. Copyright © 2020 Kmiec et al. 2020 Kmiec et al. This content is distributed under the terms of the Creative Commons Attribution 4.0 International license.

The five HIV-2 IMCs differed substantially in their susceptibility to ZAP (Fig. 2A). The primary (and hence most relevant) HIV-2 7312A strain derived from peripheral blood mononuclear cells (PBMCs) of an immunosuppressed subject (20) was essentially resistant to ZAP. In contrast, HIV-2 ROD10, which has been extensively passaged in cell lines (21), was highly susceptible to ZAP although it contains the lowest number of CpGs among all HIV-2 IMCs analyzed. On average, HIV-2 strains were significantly less susceptible to ZAP than HIV-1 (Fig. 2B) despite ∼2-fold-higher CpG frequencies (Fig. 1). Similarly, HIV-2 was less sensitive to ZAP inhibition than its simian counterpart SIVsmm (Fig. 2B) and may thus have evolved reduced ZAP sensitivity despite the acquisition of higher CpG frequencies. SIVsmm has also been transmitted to rhesus macaques, giving rise to SIVmac, which causes rapid AIDS-like disease in this nonnatural host (22, 23). Three strains of SIVmac were relatively resistant to ZAP (Fig. 2A, lower left). Finally, human ZAP inhibited SIVtan and SIVsab from tantalus and sabaeus monkeys, respectively, about as efficiently as primary HIV-1 strains. Like other antiviral restriction factors, ZAP shows evidence for positive selection pressure at least in its poly(ADP-ribose) polymerase (PARP) domain (24), and it remains to be determined whether these SIV strains are equally susceptible to the ZAP orthologs of their respective host species.

To identify possible regions in the HIV-1 genome involved in ZAP sensitivity, we performed comprehensive statistical analyses between virus CpG content and degree of its restriction by ZAP. We found that the CpG frequency in coding regions of primate lentiviruses did not correlate with their susceptibility to ZAP (Fig. 2C). Instead, we observed reduced ZAP sensitivity with increased CpG frequency for the HIV-2/SIVsmm lineage, which reached significance for the *gag* gene (Fig. 2C, lower panel). It has previously been shown that artificial increases of CpGs at the 5′ end of the *env* gene increase sensitivity to ZAP (6). Examining CpG frequencies and ZAP sensitivity throughout the HIV-1 *env* gene, we found a significant correlation between the number of CpGs in the first 1,000 nucleotides (nt) and susceptibility to ZAP (Fig. 2D). This was not observed for the remainder of HIV-1 *env* (Fig. 2D) or *env* genes of other primate lentiviruses (Fig. S2).

10.1128/mBio.02903-19.2 FIG S2 Correlation between the number of CpG dinucleotides in the indicated regions of primate lentiviral *env* genes and the infectious virus yield in the presence of ZAP. Symbols in all panels represent the average value obtained for one IMC from the indicated groups in five experiments. See the Fig. 2 legend for further detail. Download FIG S2, TIF file, 2.0 MB. Copyright © 2020 Kmiec et al. 2020 Kmiec et al. This content is distributed under the terms of the Creative Commons Attribution 4.0 International license.

### CpG frequency in the first part of the HIV-1 *env* gene correlates with ZAP sensitivity.

**FIG 3:**
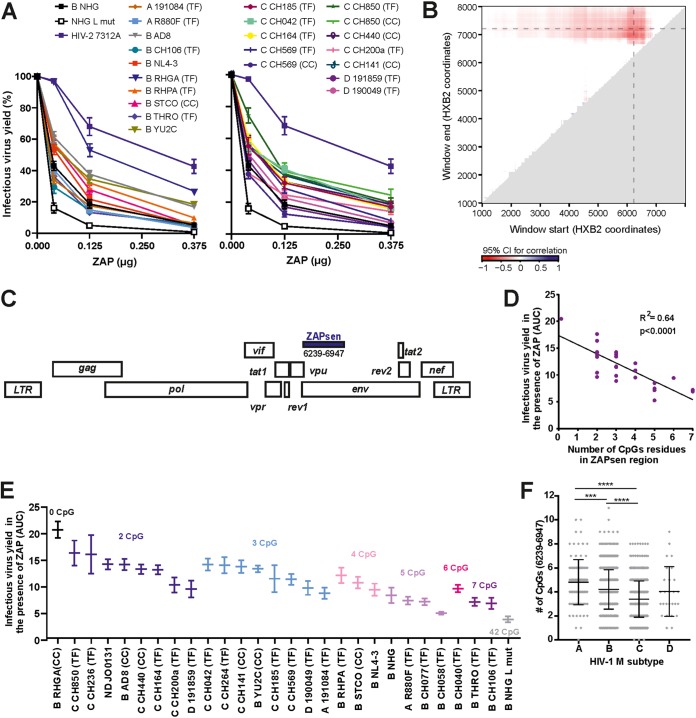
CpG numbers at the beginning of the HIV-1 *env* gene correlate with ZAP sensitivity. (A) Proviral HIV-1 constructs and increasing amounts of plasmids expressing the N-terminally HA-tagged long isoform of human ZAP were cotransfected into HEK293T ZAP KO cells. Infectious virus yield was measured using the TZM-bl reporter cell infectivity assay. For each proviral construct, values were normalized to the infectious virus yield obtained in the absence of ZAP (100%). Shown is the mean from 4 independent experiments measured in triplicates ± SEM. (B) Correlation between ZAP sensitivity and the number of CpG dinucleotides throughout HIV-1 genomes. Mapping of the correlation of ZAP sensitivity to the number of CpG dinucleotides within a region was performed using sliding window analysis. Colors indicate the boundary of the 95% confidence interval (CI) closest to 0 for the correlation (intervals overlapping *r* = 0 are set to 0) between virus yield in the presence of ZAP (measured by AUC) and the number of CpG dinucleotides within the region starting (*x* axis) and ending (*y* axis) at the given coordinate. Dashed horizontal and vertical lines indicate the boundaries of the LM region analyzed by Takata and colleagues (6). (C) Localization of the best correlation region (referred to as ZAPsen region) in HIV-1 (corresponding to nucleotides 6239 to 6947 in the HXB2 reference genome). (D) Correlation between the frequency of CpGs in the ZAPsen region and the corresponding virus yield in the presence of ZAP. (E) HIV-1 strains were sorted based on the number of CpG residues in the ZAPsen region. Shown is the average virus production as area under the curve (AUC) derived from ZAP titration. TF, transmitted-founder isolate; CC, chronic viral isolate. (F) Number of CpG dinucleotides in the ZAPsen region of *env* of HIV-1 subtype A, B, C, or D strains. Viral sequences were obtained from the Los Alamos database and analyzed for CpG content in the defined region. Significant differences were calculated using Mann-Whitney U-test. ***, *P* < 0.001; ****, *P* < 0.0001.

To further define viral determinants of ZAP sensitivity, we expanded our analyses to a panel of 29 HIV-1 group M IMCs, performing sliding window analyses to identify relevant regions in the viral genome. Our panel included subtype A, B, C, and D strains as well as IMCs of 14 transmitted-founder (TF) viruses which initiated *de novo* infection (25, 26). As expected, ZAP susceptibility of HIV-1 IMCs varied. However, all viruses tested were less sensitive than the NHG L strain, which contains artificially high levels of CpGs (6), but more sensitive than the HIV-2 7312A strain (Fig. 3A). To define the region(s) in the HIV-1 genome involved in ZAP sensitivity in an unbiased manner, we compared infectious virus yield in the presence of ZAP (measured by area under the curve [AUC]) with the number of CpGs in every possible subregion of the HIV-1 IMC genomes (Fig. 3B). The best-correlated region corresponded to nucleotides 6239 to 6947 located in the 5′ region of the *env* gene of the HIV-1 HXB2 reference genome (Fig. 3C). The number of CpGs in this region significantly correlated with the susceptibility of HIV-1 to ZAP inhibition (Fig. 3D). These differences in ZAP sensitivity and CpG numbers were not associated with increased usage of CpG-containing codons or alterations in amino acid composition (Fig. S3). Sorting the HIV-1 IMCs based on the number of CpGs in the particular *env* region revealed some differences between viruses that contained the same number of CpGs, indicating that other viral features also contribute to ZAP sensitivity (Fig. 3E). Most subtype C IMCs exhibited lower numbers of CpGs in the *env* region of interest than did other subtypes of HIV-1 (Fig. 3E). Indeed, examination of ∼1,500 sequences from the Los Alamos database confirmed that subtype C HIV-1 strains encode significantly lower numbers of CpGs in the 5′ third of *env* (3.43 ± 0.06) than subtype A (4.90 ± 0.21) and B (4.20 ± 0.06) HIV-1 strains (numbers shown as mean ± SEM) (Fig. 3F). Since the number of HIV-1 IMCs available for functional analysis was limited, the identified region ranging from nucleotides 6239 to 6947 in HXB2 represents an approximation of the most critical region for HIV-1 ZAP susceptibility. Nonetheless, our results strongly suggest that this part of the HIV-1 *env* gene represents a major determinant of ZAP sensitivity. Therefore, we refer to it as the ZAP sensitivity (ZAPsen) region here.

10.1128/mBio.02903-19.3 FIG S3 Relative synonymous codon usage (RSCU) and percent amino acid composition of tested HIV-1 strain *env* fragment corresponding to positions 6239 to 6947 of the reference HIV-1 HXB2 sequence (ZAPsen region). Codons and amino acids that contain a CpG dinucleotide are shown in brackets. RSCU is defined as the ratio of the observed frequency of codons to the expected frequency given that all the synonymous codons for the same amino acids are used equally. A codon that is used less frequently than expected will have an RSCU value of less than 1.00 (blue) and vice versa for a codon that is used more frequently than expected (red). RSCU values were calculated using the CALcal online tool (http://genomes.urv.es/CAIcal/), and percent amino acid usage was determined by the COPId online tool (http://crdd.osdd.net/raghava/copid/whole_comp.html). Download FIG S3, TIF file, 2.5 MB. Copyright © 2020 Kmiec et al. 2020 Kmiec et al. This content is distributed under the terms of the Creative Commons Attribution 4.0 International license.

### CpG numbers in ZAPsen correlate with ZAP effect on HIV-1 mRNA and protein levels.

**FIG 4:**
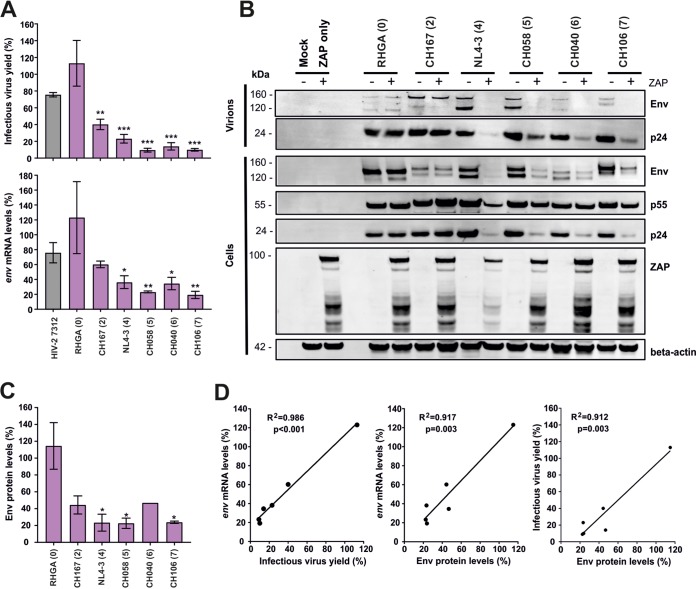
Effect of ZAP on HIV-1 RNA and protein expression. (A) Effect of ZAP on infectious HIV-1 yield (top) and the levels of viral *env* mRNA (bottom). Shown are mean values (±SEM) from at least three experiments. Numbers in parentheses indicate the number of CpGs in the ZAPsen region of each tested HIV-1 strain. Asterisks indicate significant differences from the HIV-2 7312 control construct. (B) Viral protein expression in transfected HEK293T cells and virions in the presence or absence of ZAP. To examine the effect of ZAP on viral protein expression levels, HEK293T ZAP KO cells were cotransfected with the indicated HIV-1 IMCs and ZAP expression or control vector and analyzed as described in Materials and Methods. (C) Quantification of Env protein expression levels in the presence of ZAP. Asterisks indicate significant differences from the HIV-1 RHGA control construct. (D) Correlations between infectious virus yield in the presence of ZAP and *env* mRNA or Env protein expression levels. *, *P* < 0.05; **, *P* < 0.01; ***, *P* < 0.001.

To examine the impact of CpG numbers in the ZAPsen region of *env* on the levels of viral RNA and protein expression, we cotransfected HEK293T ZAP knockout (KO) cells with various HIV-1 IMCs and ZAP expression or control vectors. As expected from our previous data, ZAP had little if any inhibitory effect on HIV-1 RHGA and the control HIV-2 7312A constructs but efficiently reduced infectious virus yield of HIV-1 strains containing higher numbers of CpGs in the ZAPsen region (Fig. 4A). Quantitative analyses (Fig. S4) showed that ZAP had a similar effect on infectious virus yield and the levels of *env* RNA transcripts (Fig. 4B). Higher numbers of CpGs in the ZAPsen region of *env* were associated with strongly reduced levels of p24 and Env expression in the presence of ZAP (Fig. 4B and C). The p55 Gag precursor was less severely affected than p24 capsid antigen, presumably because of reduced expression of the viral protease required for Gag processing. The levels of infectious virus and *env-*containing mRNA correlated with one another and with the residual Env expression levels in the presence of ZAP (Fig. 4D) (all *R*^2^ values were >0.9 and *P* values were <0.003), which is consistent with the notion that ZAP targets *env*-containing incompletely spliced HIV-1 RNA (6). These data provide further evidence that CpGs in the ZAPsen region contribute importantly to promote ZAP-dependent suppression of viral transcription, protein expression, and infectious virus production.

10.1128/mBio.02903-19.4 FIG S4 Validation of mRNA quantification assay. To validate the selected *env* primer probes, serial dilutions of known copy numbers of HIV-1 NL4-3 and HIV-2 7312 plasmids were used to generate standard curves (*n* = 2; ± SD). Download FIG S4, TIF file, 1.9 MB. Copyright © 2020 Kmiec et al. 2020 Kmiec et al. This content is distributed under the terms of the Creative Commons Attribution 4.0 International license.

### Increased CpG numbers in the ZAPsen region render HIV-1 more susceptible to ZAP.

**FIG 5:**
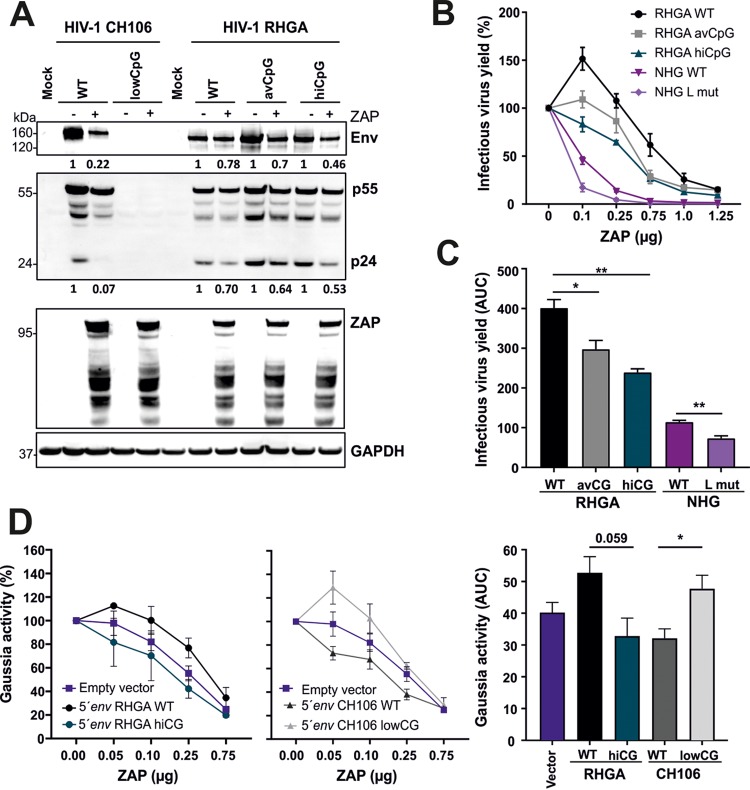
Artificial CpG enrichment of the ZAPsen region increases HIV-1 RHGA susceptibility to ZAP. (A) Viral protein expression of CpG-modified viruses in the presence or absence of ZAP. To examine the effect of ZAP on viral protein expression levels, HEK293T ZAP KO cells were cotransfected with suppressed CpG HIV-1 CH106 (low CpG) and increased CpG HIV-1 RHGA (avCpG containing 5 CpGs and hiCpG containing 10 CpGs) and ZAP expression (0.5 μg of ZAP vector DNA) or control vector and analyzed as described in Materials and Methods. (B) HEK293T ZAP KO cells were cotransfected with the indicated CpG modified proviral constructs along with increasing amounts of plasmid expressing the N-terminally HA-tagged long isoform of human ZAP. Infectious virus yield was measured using the TZM-bl reporter cell infectivity assay. For each proviral construct, values were normalized to the infectious virus yield obtained in the absence of ZAP (100%). Shown is the mean of 3 independent experiments measured in triplicates ± SEM. (C) Graph represents the average virus production in 3 independent experiments as area under the curve (AUC) ± SEM. *, *P* < 0.05; **, *P* < 0.01; ***, *P* < 0.001. (D) HEK293T ZAP KO cells were cotransfected with *Gaussia* luciferase constructs containing WT and mutant ZAPsen regions of HIV-1 RHGA and CH106 in the 3′ UTR and increasing doses of ZAP expression vector. Two days later, the *Gaussia* luciferase activities in the culture supernatants were determined. Shown are mean values (± SEM, *n* = 3) normalized to those obtained in the absence of ZAP (100%).

To examine whether the actual number of CpGs in the ZAPsen region of *env* determines ZAP sensitivity, we decreased the number of CpGs in the HIV-1 CH106 5′ *env* region by synonymous mutations from 10 to 3 and conversely increased this number in HIV-1 RHGA from 1 to 5 and 10 (Fig. S5). Unexpectedly, reduction of CpGs in the *env* gene of CH106 abrogated viral protein expression (Fig. 5A). Thus, in some cases, CpGs are essential for HIV-1 replication possibly because they are required for effective RNA splicing. In contrast, all three RHGA constructs expressed viral proteins (Fig. 5A) and yielded comparable quantities of infectious HIV-1 in the absence of ZAP. However, HIV-1 RHGA constructs containing increased numbers of CpGs in the ZAPsen region exhibited more profound differences in Env and p24 protein expression in the presence of ZAP (Fig. 5A). Consistent with this, ZAP inhibited the HIV-1 RHGA IMCs containing 5 CpGs or 10 CpGs more severely than the parental virus (Fig. 5B and C). However, all three RHGA constructs were less susceptible to ZAP than the NHG HIV-1 strain. Thus, increased numbers of CpGs in the ZAPsen region enhance HIV-1 susceptibility to ZAP, but the viral backbone also plays a role.

10.1128/mBio.02903-19.5 FIG S5 Sequence alignment of parental and mutant HIV-1 CH106 and RGHA IMCs. Shown are nucleotide substitutions introduced into the HIV-1 CH106 and HIV-1 RHGA proviral sequence. Dots indicate nucleotide identity. Download FIG S5, TIF file, 2.1 MB. Copyright © 2020 Kmiec et al. 2020 Kmiec et al. This content is distributed under the terms of the Creative Commons Attribution 4.0 International license.

To determine whether CpGs in the first part of HIV-1 *env* genes may directly affect RNA stability in the presence of ZAP, we introduced the 5′ *env* WT and mutant regions of HIV-1 RHGA and CH106 into the 3′ untranslated region (UTR) downstream of the *Gaussia* luciferase gene. Transfection of HEK293T cells with these *Gaussia* reporter vectors and the ZAP expression construct revealed that increasing the number of CpGs in the ZAPsen region of RHGA *env* from 1 to 10 reduced luciferase expression in the presence of ZAP (Fig. 5D). Vice versa, reduction of CpGs in the HIV-1 CH106 *env* region from 10 to 3 reduced susceptibility to ZAP inhibition. These results suggest that CpGs in the ZAPsen region of *env* affect RNA stability independently of additional viral sequences.

### ZAP-sensitive *env* genes increase the susceptibility of SIVmac and HIV-2 to ZAP.

**FIG 6:**
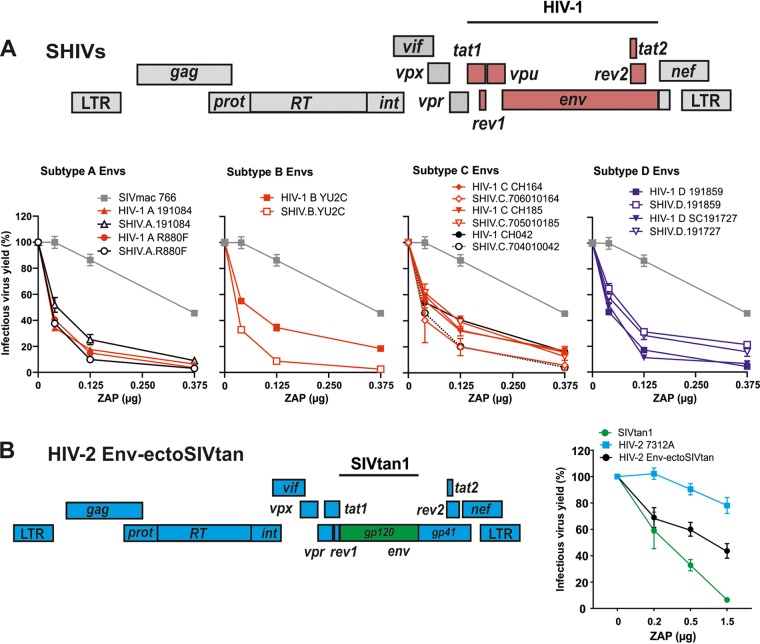
Susceptibility of chimeric primate lentiviruses to ZAP inhibition. (A) Schematic representation of the chimeric constructs between SIVmac (gray) and HIV-1 (red) described by Li and colleagues (27) (top). The indicated proviral constructs and increasing amounts of plasmid expressing the N-terminally HA-tagged long isoform of human ZAP were cotransfected into HEK293T ZAP KO cells. Infectious virus yield was measured using the TZM-bl reporter cell infectivity assay. For each proviral construct, values were normalized to the infectious virus yield obtained in the absence of ZAP (100%). (B) Genomic organization of the chimera between HIV-2 7312A (blue) and SIVtan1 (green) and susceptibility of the chimeric and parental IMCs to ZAP inhibition. Shown are the means from 3 independent experiments measured in triplicates ± SEM.

Despite higher frequencies of genomic CpGs (Fig. 1), HIV-2 and SIVmac are on average less susceptible to ZAP than HIV-1 (Fig. 2). To further determine the role of the *env* gene in primate lentiviral susceptibility to ZAP inhibition, we took advantage of a collection of chimeric simian-human viral constructs (SHIVs), in which the *env* gene and adjacent regions of SIVmac were replaced with corresponding regions of different primary HIV-1 group M strains (Fig. 6A, top) (27). All SHIV constructs were substantially more susceptible to ZAP inhibition than the parental SIVmac766 construct (Fig. 6A, bottom). The chimeric viruses showed ZAP sensitivity profiles that were highly similar to those of the corresponding HIV-1 IMCs, although in some cases they were slightly more (B YU2C) or less (D 191859 and 191727) sensitive. However, these SHIVs also contain the *tat*, *rev*, and *vpu* genes of HIV-1 (Fig. 6A, top). To more specifically examine the role of *env*, we utilized a chimeric construct containing a large fragment of the *env* gene of the ZAP-sensitive SIVtan1 strain in the backbone of the ZAP resistant HIV-2 7312A provirus (Fig. 6B, left) (28). Exchange of the *env* coding region did not reduce infectious virus yield in the absence of ZAP (Fig. S6). However, introduction of the SIVtan1-derived *env* substantially increased the susceptibility of HIV-2 7312A to ZAP (Fig. 6B, right).

10.1128/mBio.02903-19.6 FIG S6 Virus production by wild-type and chimeric viral constructs. HEK293T ZAP KO cells were transfected with the indicated HIV and SIV proviral constructs and pCG IRES BFP HA-ZAP or empty vector. Supernatants were harvested 2 days later, and infectious virus yield was determined by infecting TZM-bl indicator cells. Shown are average values ± SEM (*n* = 3) for infectious virion yield measured in three independent experiments, each with triplicate infections. Download FIG S6, TIF file, 2.0 MB. Copyright © 2020 Kmiec et al. 2020 Kmiec et al. This content is distributed under the terms of the Creative Commons Attribution 4.0 International license.

### Endogenous ZAP expression inhibits primary HIV-1 strains.

**FIG 7:**
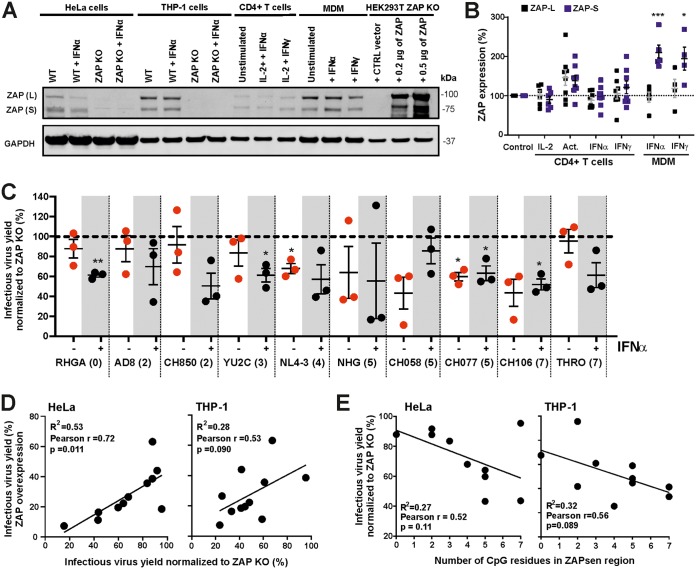
Expression and antiretroviral effect of endogenous ZAP. (A) Representative Western blot of ZAP-L and -S expression levels in HeLa, THP-1, and primary CD4^+^ T cells and monocyte-derived macrophages (MDM) or HEK293T ZAP KO cells transfected with the indicated amounts of ZAP expression or empty control vector. Cells were stimulated with 500 U/ml IFN-α or 200 U/ml IFN-γ (with or without IL-2) for 3 days or left untreated. (B) Quantification of ZAP-L and -S expression levels in primary CD4^+^ T cells and MDM from different blood donors. CD4^+^ T cells were treated with IFN as described for panel A or treated with IL-2 and anti-CD3/CD28 beads (Act.). *, *P* < 0.05; ***, *P* < 0.001. (C) Inhibitory effects of endogenous ZAP in HeLa cells transduced with HIV-1 strains in the presence or absence of IFN-α. Infectious virus yield from control HeLa cells expressing ZAP, measured 3 days postransduction by a TZM-bl reporter assay, was normalized to that obtained from HeLa ZAP KO cells (100%). Numbers in parentheses indicate the number of CpG dinucleotides in the ZAPsen region of each HIV-1 strain. Shown are the results obtained in three independent experiments, each performed in triplicate. Each dot represents the mean value from technical triplicates, with bars indicating means from three independent experiments (± SEM). (D) Correlation between the infectious virus yields in the presence of ZAP overexpression in HEK293T or endogenous ZAP expression in HeLa or THP-1 cells. (E) Correlation between the number of CpG dinucleotides in the ZAPsen region of the HIV-1 *env* gene and the infectious virus yield in the presence of ZAP in HeLa or THP-1 cells. Symbols in panels D and E represent the average value obtained for one IMC in at least three independent experiments, and infectious virus yields were normalized to those obtained in the absence of ZAP (100%).

To map the region in the HIV-1 genome determining ZAP sensitivity, we used overexpression conditions since this required a strong and robust phenotype. Western blot analysis confirmed that the ZAP expression levels in transiently transfected HEK293T cells are substantially higher than the endogenous levels of ZAP expression in cell lines or primary HIV-1 target cells (Fig. 7A). Treatment with type I interferons (IFNs) had only marginal effects on ZAP expression in primary CD4^+^ T cells but significantly enhanced expression of the short isoform of ZAP in macrophages (Fig. 7B). To determine whether endogenous ZAP restricts primary HIV-1 strains, we tested a panel of 11 HIV-1 constructs in parental and ZAP KO HeLa and THP-1 cells. Notably, the endogenous levels of ZAP expression in these cell lines were similar to those detected in primary CD4^+^ T cells and macrophages (Fig. 7A). On average, the presence of ZAP reduced the infectious HIV-1 yield in HeLa and THP-1 cells by 25.5% ± 6.2% and 49.1% ± 6.3%, respectively (mean values ± SEM) (Fig. 7C and Fig. S7A and B). Altogether, HIV-1 constructs that were highly sensitive to ZAP overexpression were also more sensitive to endogenous ZAP expression, with the reduction of infectious virus yield ranging from 4% to 80% (Fig. 7D). Thus, higher numbers of CpGs in the ZAPsen region of *env* were usually associated with increased susceptibility of HIV-1 to inhibition by endogenous ZAP (Fig. 7E and Fig. S7C). However, in contrast to the results obtained in transfected HEK293T cells, the correlations failed to reach significance, most likely because the number of HIV-1 constructs analyzed was limited and the inhibitory effects were less robust than those obtained in ZAP overexpression assays.

10.1128/mBio.02903-19.7 FIG S7 Infectious HIV-1 production in WT and ZAP KO cells. (A) Infectious virus production by IFN-α-treated (500 U/ml) or untreated parental or ZAP KO HeLa cells transfected with the indicated HIV-1 proviral constructs. Numbers in brackets indicate the number of CpGs in the ZAPsen region. Infectious virus yield was determined by TZM-bl infection assay. Each symbol represents the average value obtained from triplicate infections in one independent experiment. Bars indicate the mean values (± SEM) derived from three independent experiments. *, *P* < 0.05; **, *P* < 0.01; ***, *P* < 0.001. (B) Infectious virus production by parental and ZAP KO THP-1 cells determined as described for panel A. (C) Pairwise comparison of infectious virus yield in parental and ZAP KO HeLa (left) and THP-1 (right) cells. Symbols represent the mean values from three independent experiments, each performed in triplicate. (D) Correlation between the number of CpG dinucleotides in the ZAPsen regions of the HIV-1 *env* genes and the infectious virus yield in the presence of ZAP in HeLa cells treated with IFN-α. Values were normalized to those obtained in ZAP KO cells (100%). Download FIG S7, TIF file, 2.8 MB. Copyright © 2020 Kmiec et al. 2020 Kmiec et al. This content is distributed under the terms of the Creative Commons Attribution 4.0 International license.

We also investigated the effect of IFN-α in parental and ZAP KO HeLa and THP-1 cells lines. IFN-α treatment of THP-1 cells frequently reduced the infectious virus yield to background levels, precluding meaningful comparisons. In HeLa cells, IFN-α treatment reduced infectious HIV-1 production 4.2-fold ± 0.7-fold in the presence and 3.8 fold ± 0.6-fold in the absence of ZAP (Fig. S7A). Lack of a significant difference agrees with our finding that IFN-α does not induce ZAP expression in HeLa cells (Fig. 7A). IFN-α treatment abolished the correlation between the number of CpGs in the ZAPsen region of *env* and the infectious virus yield in the presence of ZAP (Fig. S7D), suggesting that other antiviral factors play more dominant roles in restricting HIV-1 and mask inhibitory effects of endogenous ZAP in the presence of type I IFNs. Altogether, however, our data show that endogenous levels of ZAP expression are sufficient to restrict primary HIV-1 strains and further support the idea that the number of CpGs in the ZAPsen region affects viral susceptibility to ZAP inhibition.

### Predicted structural stability of the 5′ *env* region correlates with ZAP sensitivity of HIV-1.

**FIG 8:**
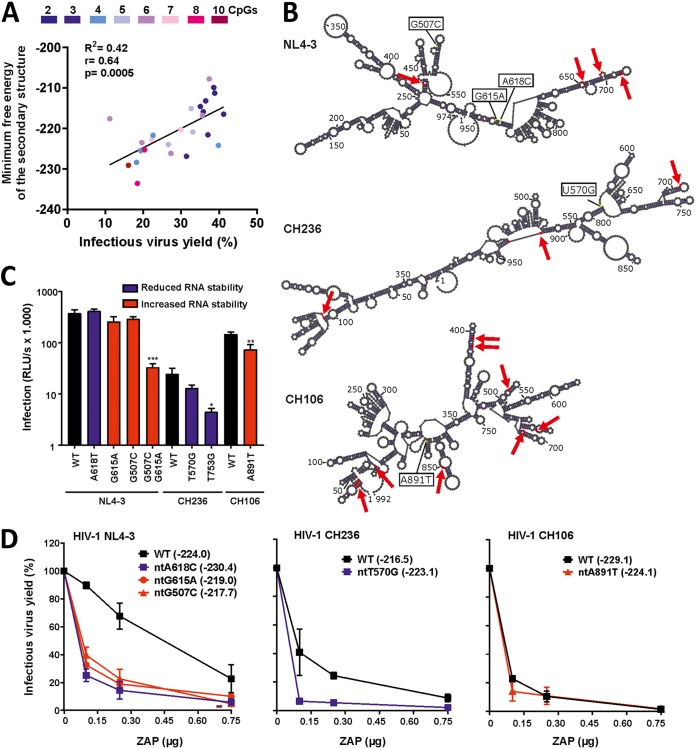
Structural features of the first part of *env* and ZAP sensitivity. (A) Correlation between the minimum free energies of the predicted RNA secondary structures of the ZAPsen region of HIV-1 *env* genes and viral susceptibility to ZAP inhibition. (B) Predicted secondary RNA structures of the HIV-1 NL4-3, CH236, and CH106 5′ *env* regions. Arrows indicate the positions of CpG dinucleotides. The localization of specific nucleotide changes analyzed is also indicated. (C) Infectious virus production by HIV-1 constructs containing changes predicted to alter the RNA stability of the ZAPsen region of *env* in the absence of ZAP expression. HEK293T ZAP KO cells were transfected with the indicated proviral HIV-1 constructs, and infectious virus yield was measured by the TZM-bl reporter assay. Results show mean values (± SEM) from three to four independent experiments, each with triplicate measurements. *P* values indicate difference from the corresponding WT HIV-1 construct: *, *P* < 0.05; **, *P* < 0.01; ***, *P* < 0.001. (D) Effect of mutations predicted to reduce (blue) or enhance (red) RNA stability on ZAP sensitivity. HEK293T ZAP KO cells were cotransfected with the indicated HIV-1 proviral constructs and increasing doses of ZAP expression vector, and infectious virus yield was measured by the TZM-bl reporter assay. Mean infection values (± SEM) were obtained from three or four independent experiments and normalized to those obtained in the absence of ZAP expression (100%).

To obtain first insights into the mechanism underlying the relevance of the ZAPsen region, we performed secondary structure prediction analyses of the beginning of the 5′ region of the *env* encoding RNA encompassing the ZAPsen region. We found a highly significant correlation between the minimum free energy of the predicted RNA structures of this part of the *env* gene and HIV-1 ZAP sensitivity (Fig. 8A). To experimentally examine whether alterations in structural feature may impact ZAP sensitivity, we introduced specific nucleotide changes into the HIV-1 NL4-3, CH236, and CH106 *env* genes (Fig. 8B). The selected nucleotide substitutions were predicted to significantly alter the minimum free energy of the 5′ *env* secondary structure without changing known splice sites, Env translation, and the number of CpG dinucleotides. Alterations of G507C/G6515A in NL4-3 and T753G in CH236 impaired infectious virus production independently of ZAP expression (Fig. 8C), hampering meaningful analysis. All remaining substitutions increased the susceptibility of HIV-1 NL4-3, CH236, and CH106 to ZAP inhibition, irrespectively of whether the nucleotide changes predicted reduction or increase of the minimum free energy of the secondary RNA structures (Fig. 8D). These results suggest that both the number of CpGs and the RNA secondary structure of the beginning of the *env* gene affect HIV-1 susceptibility to ZAP inhibition, although the exact determinants remain to be clarified.

### Correlation between CpG numbers in the ZAPsen region and disease progression.

**FIG 9:**
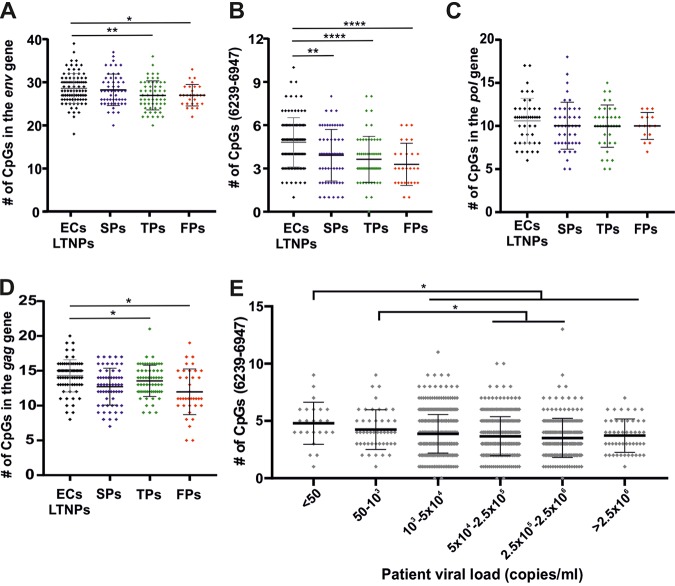
CpG frequencies and disease progression. (A to D) Number of CpG dinucleotides in the *env* gene (A), ZAPsen region of *env* (B), *pol* gene (C), and *gag* gene (D) of HIV-1 strains from individuals showing different rates of disease progression (EC, elite controller; LTNP, long-term nonprogressor; SP, TP, and FP, slow, typical, and fast progressor, respectively). Single viral sequences of HIV-1 patients were obtained from the Los Alamos database, analyzed for CpG content in the defined region, and plotted against the patient’s disease progression type as reported by the sequence-submitting authority. (E) CpG number in the ZAPsen region of HIV-1 strains grouped based on the viral load in the untreated infected individuals. Significant differences were calculated using the Mann-Whitney U-test. *, *P* < 0.05; **, *P* < 0.01; ****, *P* < 0.0001.

It has been reported previously that higher CpG dinucleotide frequency in the HIV-1 *env* gene is associated with lower disease progression rates (29). However, only six slow and 12 “typical” progressors were examined, and only sequences corresponding to the C2-V5 region (residues 6813 to 7634 in HXB3) of HIV-1 *env*, which only partially overlaps the ZAPsen region, were analyzed. We thus took advantage of viral sequences from individuals with reported progression rates available in the Los Alamos HIV database. We examined sequences from 243 HIV-1-infected individuals (one viral sequence from each person), representing 95 elite controllers/long-term nonprogressors (EC/LTNP), 60 slow progressors (SP), 60 typical progressors (TP), and 28 rapid progressors (RP). We found that the average number of CpGs in the full-length *env* gene was slightly higher in HIV-1 strains from EC/LTNP than those from individuals with progressing HIV-1 infection (Fig. 9A). However, the differences in CpG numbers became more pronounced when we focused on the ZAPsen region for calculation (Fig. 9B). The average numbers of CpGs declined from 4.81 ± 0.17 in EC/LTNP, over 3.92 ± 0.23 in SPs and 3.63 ± 0.21 in TPs, to 3.29 ± 0.28 in RPs (numbers give mean values ± SEM). No significant differences between progression groups were observed for the number of CpGs in the *pol* open reading frame (ORF) (Fig. 9C). Similarly, the average number of CpGs in the *gag* gene was slightly higher in HIV-1 sequences from EC/LTNP than in progressing individuals (Fig. 9D), although it has been reported previously that this increased CpG abundance may decrease HIV-1 replication by several ZAP-independent mechanisms (30). We also examined possible correlations between CpG numbers and viral loads. Although the number of CpGs in the ZAPsen region varied substantially (Fig. 9E), significantly higher numbers of CpGs were found in HIV-1 strains from individuals with low viral loads than in those with high viral loads. Thus, the number of CpG dinucleotides in the ZAPsen region of *env* appears to be linked to viral replication and disease progression, although the mechanisms involved remain to be characterized.

## DISCUSSION

In the present study, we show that CpG dinucleotides are generally suppressed in lentiviral genomes, albeit to an unexpectedly different extent. We also demonstrate that the overall frequency of CpG dinucleotides in proviral HIV and SIV genomes does not correlate with viral susceptibility to ZAP inhibition. However, analysis of a large number of infectious molecular clones representing transmitted-founder and chronic HIV-1 strains revealed that the number of CpG residues in an ∼700-nucleotide region at the 5′ end of the *env* gene correlates with viral susceptibility to ZAP. Specific mutant HIV-1 and recombinant chimeric viral constructs confirmed that the number of CpG dinucleotides in the ZAPsen region of *env* is an important determinant of ZAP sensitivity. We further identified a significant correlation between the minimum free energy of the predicted RNA secondary structures of the 5′ end of the various *env* genes and ZAP sensitivity of the corresponding HIV-1 molecular clones. Our results show that the number of CpGs in the first part of the HIV-1 *env* gene is a key determinant of ZAP sensitivity but that other factors, including the secondary RNA structure of this region, also play a role.

Our results show that the frequency of CpG dinucleotides in the viral genome changed after cross-species transmission of primate lentiviruses. For example, SIVgsn/mus/mon strains exhibit higher CpG frequencies throughout their genomes than SIVcpz (Fig. 1), which resulted from a recombination event between the precursor of these SIVs and other monkey viruses (16, 19). Similarly, HIV-2 exhibits a 2-fold-higher CpG content than SIVsmm, indicating a significant increase after zoonotic transmission. Further studies are required to elucidate the driving forces behind these changes and their effects on viral replication. It has been reported that the short isoform of ZAP can be induced by IFN in HEK293T and HeLa cells (31, 32). Thus, the selective pressures to counteract ZAP might be higher in pathogenic infection associated with higher levels of immune activation and inflammatory cytokine expression. In agreement with this possibility, pathogenic HIV-2 and SIVmac strains are less sensitive to ZAP inhibition than SIVsmm, which replicates to high titers in naturally infected sooty mangabeys without causing disease (33, 34). Interestingly, HIV-2 is less susceptible to ZAP than HIV-1 and SIVsmm despite significantly higher CpG content. It will thus be important to determine whether HIV-2 evolved effective ZAP evasion mechanisms during human adaptation. Further studies seem also warranted in the case of SIVs infecting *Cercopithecus* monkeys. It is currently unknown whether these viruses cause disease, but their low prevalence suggests suboptimal adaptation to their hosts (35). These viruses exhibit the highest genomic CpG frequencies of all primate lentiviruses, and it will thus be interesting to determine whether they can evade or antagonize ZAP-mediated restriction in their primate hosts.

We found that the number of CpG residues in the 5′ third of the viral *env* gene was highly predictive of the susceptibility of HIV-1 to ZAP-mediated restriction. No such correlation between CpG numbers in the corresponding region of *env* and susceptibility to ZAP inhibition was observed for HIV-2 and the various SIVs. Thus, the determinants of ZAP sensitivity of HIV-1 seem to be distinct from those of other primate lentiviral lineages. It remains to be determined why this HIV-1 *env* region seems to be a particularly important ZAP target. One possible explanation is the presence of a *vpu* gene in HIV-1. *env* and *vpu* are both produced via leaky scanning of the same bicistronic RNA. Thus, it is tempting to speculate that the levels of Env expression by HIV-1 are lower than those achieved by non-Vpu-encoding primate lentiviruses. It has been established that HIV-1 particles contain only a low number of Env trimers (about 7 to 11 spikes) (36, 37). Thus, HIV-1 might be particularly susceptible to further reductions in Env expression due to the degradation of single-spliced or unspliced HIV-1 RNAs. We have previously shown that increased Env expression due to *vpu* inactivation allows HIV-1 to partly evade restriction by GBP5, which impairs virion infectivity by interfering with furin-mediated Env processing (38–40). However, data obtained using a chimeric virus that differs only in the *env* coding region (Fig. 6B) suggest that *env* affects ZAP sensitivity independently of the presence of a *vpu* gene.

We found that some synonymous nucleotide changes at the beginning of the *env* gene severely impaired infectious HIV-1 production in the absence of ZAP (Fig. 8C). The reasons for these disruptive effects remain to be determined, but it has recently been reported that the 5′ region of HIV-1 *env* RNA transcripts contains important functional and structural elements (41). Most interestingly, several single nucleotide changes in the 5′ regions of HIV-1 NL4-3 and CH236 *env* genes clearly increased ZAP sensitivity without altering the number of CpGs or reducing infectious virus yield in the absence of ZAP (Fig. 8). We found a significant correlation between the minimum free energy of the predicted RNA structures of the 5′ region of primary HIV-1 *env* genes and ZAP sensitivity (Fig. 8A). Notably, ∼70% of CpGs in the predicted structures were unpaired (data not shown). Thus, it was tempting to speculate that a more stable secondary structure of this *env* region might increase accessibility of CpGs and ZAP sensitivity. However, artificial point mutations in the 5′ region increased HIV-1 susceptibility to ZAP inhibition, irrespectively of whether they predicted reduction or increase of the minimum free energy of the secondary RNA structures. Altogether, these results suggest that not only the number of CpGs but also their exposure, localization, and structural environment affect ZAP sensitivity of HIV-1. The mutant HIV-1 NL4-3 and CH236 constructs described in the present study might be useful tools to further define the structural determinants of ZAP sensitivity in the future.

It has been previously shown that ZAP efficiently inhibits HIV-1 NHG constructs containing artificially high levels of CpG dinucleotides but not the parental wild-type virus (6). To map viral determinants of ZAP sensitivity, we overexpressed ZAP and found that at high levels it efficiently inhibits essentially all primary HIV-1 IMCs, although most of them were less susceptible to ZAP than the HIV-1 NHG strain (Fig. 2). The levels of ZAP expression in transiently transfected HEK293T cells were higher than those observed in primary CD4^+^ T cells and macrophages representing the major viral target cells *in vivo*. They are highly similar, however, to the levels of endogenous ZAP expression in HeLa and THP-1 cells (Fig. 7A). ZAP knockout almost invariantly increased infectious virus yield, particularly if the HIV-1 molecular clones contained relatively high numbers of CpGs in the ZAPsen region of the *env* gene. These results suggest that the observed differences in viral sensitivity to ZAP overexpression may be relevant for HIV-1 replication in primary viral target cells. Examination of HIV-1 sequences from individuals with known rates of clinical progression supports a role of the number of CpGs in the ZAPsen region of *env* in clinical progression to AIDS. Whether reduced sensitivity to ZAP or other effects of reduced CpG numbers are responsible for this link to disease progression has to be addressed in future studies.

Most HIV-1 strains have between 3 and 6 CpGs in the ZAPsen *env* region and are sensitive to high expression levels of ZAP. In contrast, the HIV-1 RHGA strain encodes no CpG dinucleotides in this region and was resistant to ZAP inhibition. To examine whether the lack of CpGs in this region was directly responsible for ZAP resistance, we generated mutants of HIV-1 RHGA that contained increased numbers of CpGs. Our results showed that higher numbers of CpGs in the ZAPsen region increased the susceptibility of HIV-1 to ZAP inhibition. Analysis of yet-to-be-defined additional structural and functional elements may explain why HIV-1 does not eliminate all CpG dinucleotides in this region and why removal of CpGs from the HIV-1 CH106 strain rendered this virus nonfunctional. Notably, ZAP itself does not degrade viral RNA and needs TRIM25 and KHNYN as cofactors for efficient restriction (42, 43). KHNYN contains an RNase NYN domain and seems to be critical for viral RNA degradation (44), while the role of TRIM25 remains to be determined. In this study, we focused on the inhibitory effects of ZAP. Further analyses of the interplay between ZAP, TRIM25, and KHNYN in restricting HIV-1 and other lentiviruses will be of significant interest.

## MATERIALS AND METHODS

### Cell lines.

HEK293T ZAP KO cells have been previously described (6). HeLa ZAP CRISPR KO (ZAP-G1) and a control cell line, HeLa control cells with CRISPR targeting the firefly luciferase gene (control *luc*), have been previously described (44). TZM-bl cells, which express CD4, CCR5, and CXCR4 and contain the β-galactosidase genes under the control of the HIV promoter (45, 46), have been kindly provided by J. C. Kappes and X. Wu and Tranzyme Inc. through the NIH AIDS Reagent Program. Cell lines were cultured in Dulbecco’s modified Eagle’s medium (DMEM) supplemented with 2.5% (during virus production) or 10% (at all other times) heat-inactivated fetal calf serum (FCS), 2 mM l-glutamine, 100 U/ml penicillin, and 100 μg/ml streptomycin. THP-1 CRISPR ZAP KO and their control THP-1 monocytic cell line were previously described (47). THP-1 cell lines were cultured in RPMI supplemented with 10% FCS, 2 mM l-glutamine, 100 U/ml penicillin, and 100 μg/ml streptomycin. Prior to usage, THP-1 cells were treated with Mynox Gold (Minerva Biolabs). Cell lines were regularly tested and confirmed to be mycoplasma negative.

### Expression constructs.

The vector encoding human ZAP-L containing an N-terminal hemagglutinin (HA) tag was generated by cloning human ZAP (GenScript ORF cDNA clone no. OHu25350) into XbaI/MluI sites of the pCG internal ribosome entry site (IRES) blue fluorescent protein (BFP) plasmid. The sequence of the insert was confirmed to match the NCBI reference sequence NM_020119.4. Primate lentiviral IMCs and their derivatives have been described previously, and their accession numbers are provided below.

### Cell viability assay.

To determine the effects of ZAP overexpression on cell viability, HEK293T ZAP KO cells were transfected with increasing concentrations of pCG HA-ZAP IRES BFP expression plasmid (0.1, 0.5, or 2.5 μg per one million cells) and normalized with pCG IRES BFP vector. After 48 h, the CellTiter-Glo substrate (catalog no. G7570; Promega) was added to the transfected cells. Luminescence, which correlates with the number of viable cells, was measured using a plate reader.

### Lentiviral sensitivity to ZAP.

To assess relative lentiviral sensitivity to ZAP overexpression, HEK293T ZAP KO cells (in 24-well format) were cotransfected using polyethyleneimine (PEI) transfection reagent with 625 ng of the indicated IMC and increasing concentrations (0, 40, 125, and 375 ng) of pCG HA-ZAP IRES BFP expression vector and the amount of DNA was normalized to 1 μg by adding pCG IRES BFP vector. Virus-containing supernatants were harvested 2 days later and used to infect TZM-bl reporter cells in triplicates. β-Galactosidase activity was measured 2 days later using the Gal-Screen kit (Applied Biosystems) as relative light units per second using a microplate luminometer. Infectious virus yield values of each IMC in the presence of ZAP were normalized to the corresponding BFP-only control. Linear correlations between sensitivity to ZAP, calculated as area under the curve (AUC), and CpG frequency, calculated as a ratio of CpG number in a given viral region to the length of the sequence, were generated using GraphPad Prism software.

### Lentiviral sequence analysis and CpG mapping.

Sequences representative of different primate lentiviral lineages were obtained from the NCBI database (https://www.ncbi.nlm.nih.gov/nuccore). A cladogram showing the relationship between these lentiviruses was generated using the ClustalW2 simple phylogeny tool (https://www.ebi.ac.uk/Tools/phylogeny/simple_phylogeny/) and is based on the first 6,000 nt of the viral sequence. For the purpose of showing genomic CpG distribution, lentiviral sequences were aligned (https://www.ebi.ac.uk/Tools/msa/) and the CpG number per 100-bp window of each aligned sequence was determined using a custom-written Python script. Frequencies of CpG nucleotide in the coding viral genome region or an indicated part of the genome were calculated as a ratio of CpG number to the length of the analyzed sequence.

### qRT-PCR.

*env* transcript levels were determined in HEK293T ZAP KO cells cotransfected in a 6-well dish with 2.5 μg of the indicated IMC and 0.5 μg of pCG IRES BFP vector encoding ZAP-L or BFP only. At 40 h posttransfection, cells were washed with PBS and lysed in RLT Plus buffer containing 1% β-mercaptoethanol. Total RNA was isolated using the RNeasy Plus minikit (catalog no. 74136; Qiagen) according to the manufacturer’s instructions. Residual genomic DNA was removed from the RNA preparations using the DNA-Free DNA removal kit (catalog no. AM1906; ThermoFisher). RNA concentrations were determined using the NanoDrop 2000 spectrophotometer, and for each sample, equal RNA amounts were subjected to cDNA synthesis using the PrimeScript RT reagent kit (catalog no. RR037A; TaKaRa) with random 6-mers and oligo(dT) primers. Reaction mixtures without reverse transcriptase were included as controls to exclude contamination with genomic DNA. cDNA was used for qRT-PCR using TaqMan Fast Universal PCR master mix (catalog no. 4352042; ThermoFisher), and viral primer-probe sets were used in multiplex reactions with glyceraldehyde-3-phosphate dehydrogenase (GAPDH) (catalog no. Hs99999905_m1; ThermoFisher) as control. HIV primers/probes were designed as follows: HIV-1 subtype B Env, CAGCAGGAAGCACTATGGGCGCA, using the forward primer CAAARAGAAGAGTGGTGCARAGAG and reverse primer GCCTCAATAGCYCTCAGC; probe for HIV-2 Env, ATACTTGGGGAACCATACAGTGCWWGCCA, using forward primer CATTCCCCTCTTTTGTGCAAC and reverse primer CATCTTCTAYTGCYTGTTCTGTTACTG. Threshold cycle (*C_T_*) data were processed relative to the GAPDH control and further normalized to control cells transfected with the indicated IMC and pCG IRES BFP control vector.

### Western blotting.

To examine the viral protein levels under the expression of ZAP, HEK293T ZAP KO cells were cotransfected in 12-well plates with 1.25 μg of indicated IMC and 0.25 μg DNA of pCG HA-ZAP IRES BFP vector or empty pCG IRES BFP vector. Two days posttransfection, cells were lysed with coimmunoprecipitation (co-IP) buffer (150 mM NaCl, 50 mM HEPES, 5 mM EDTA, 0.10% NP-40, 0.5 mM sodium orthovanadate, 0.5 mM NaF, protease inhibitor cocktail from Roche), and cell-free virions were purified by centrifugation of cell culture supernatants through a 20% sucrose cushion at 20,800 × *g* for 90 min at 4°C and lysed in co-IP lysis buffer. Samples were reduced in the presence of β-mercaptoethanol by boiling at 95°C for 10 min. Proteins were separated in 4% to 12% Bis-Tris gradient acrylamide gels (Invitrogen), blotted onto a polyvinylidene difluoride (PVDF) membrane, and incubated with anti-HIV-1 Env (catalog no. 12559; obtained through the NIH AIDS Reagent Program), anti-p24 (catalog no. ab9071; Abcam), anti-HA tag (catalog no. C29F4; Cell Signaling), anti-GAPDH (catalog no. sc-365062; Santa Cruz), and anti-ZAP (catalog no. GTX120134; GeneTex) antibodies. Subsequently, blots were probed with IRDye 680RD goat anti-rabbit IgG(H+L) (catalog no. 926-68071; LI-COR) and IRDye 800CW goat anti-mouse IgG(H+L) (catalog no. 926-32210; LI-COR) Odyssey antibodies and scanned using a Li-Cor Odyssey reader.

### Generation of CpG mutant HIV-1 constructs.

To generate proviral constructs differing specifically in CpG numbers in the first part of the *env* gene, mutant HIV-1 *env* sequences of interest were chemically synthesized (BaseClear). The synthesized sequences were introduced into the proviral HIV-1 RHGA and CH106 IMCs using the single XbaI and BbvCI or two MfeI restriction sites, respectively. The presence of the desired mutations and absence of additional changes were confirmed by Sanger sequencing. To mutate HIV-1 RHGA, we aligned HIV-1 subtype B *env* sequences found in the Los Alamos database and introduced additional CpGs at positions where CpGs are commonly present in other HIV-1 strains. Mutations in RHGA and CH106 did not change the predicted amino acid sequence of Env, and no new AG or GU dinucleotides were introduced to avoid the creation of new splice donor or acceptor sites.

### Analysis of CpG numbers in patient sequences.

Single sequences from patients with submitter-defined disease progression type or viral loads were obtained from the Los Alamos HIV Sequence Database (https://www.hiv.lanl.gov/content/sequence/HIV/mainpage.html), and the number of CpG dinucleotides in different viral genome regions was counted. Only drug-naive patient sequences were included in the viral load/CpG number analysis.

### Sliding window analysis.

The IMC genomes were aligned to the HXB2 reference genome using the Los Alamos HIV sequence database HIValign (HMM-align option). All unique nucleotide positions containing a CpG were elucidated, and the numbers of CpGs within each sequence in all possible comparisons of start and end regions were counted using R v3.4.4 (https://www.r-project.org/). Comparisons of correlations were performed using a test of difference between two overlapping correlations of dependent groups.

### Transduction of THP-1 and HeLa cells.

Vesicular stomatitis virus glycoprotein (VSV-g)-pseudotyped virus stocks were prepared by transfecting HEK293T cells with 1 μg VSV-g and 5 μg proviral construct, followed by a medium change. Supernatants were harvested 48 h later and normalized based on infectivity as measured by the TZM-bl reporter assay. THP-1 and HeLa cells were treated with IFN-α (500 U/ml) 2 days before transduction or left unstimulated. HeLa and THP-1 ZAP KO and WT cell lines were transduced with VSV-g-pseudotyped viruses. The input virus was removed by repeated washing in PBS. Three days later, cell supernatants were harvested and used to infect TZM-bl reporter cells in triplicates. β-Galactosidase activity was measured 2 days later using the Gal-Screen kit (Applied Biosystems) as relative light units per second using a microplate luminometer.

### Stimulation of primary cells.

Macrophages were derived from human PBMCs as described previously (38). CD4^+^ T cells isolated from human PBMCs (Biocoll separating solution 0396F) were stimulated with interleukin-2 (IL-2) (10 ng/ml) plus IFN-α (500 U/ml) or IL-2 (10 ng/ml) plus IFN-γ (200 U/ml) or left unstimulated. Three days poststimulation, cells were harvested and protein expression was analyzed by Western blotting.

### *Gaussia* luciferase activity.

To investigate the ability of ZAP to recognize and degrade HIV *env* sequences, 5′ *env* regions of HIV-1 CH106 from 1 to 966 nt (WT and CpG low), and RHGA from 1 to 906 nt (WT, CpG average and CpG high), were cloned into the 3′ UTR downstream of the *Gaussia* luciferase gene of a pCMV vector (Thermo Scientific catalog no. 16147). Two nanograms of the *env Gaussia* luciferase constructs was cotransfected in triplicates into HEK293T ZAP KO cells in 96-well plates, with increasing concentrations of pCG HA-ZAP IRES BFP (0, 0.025, 0.05, 0.1, 0.25, and 0.75 μg). pCG IRES BFP empty vector was added to normalize the transfected DNA amount to 2 μg. At 48 h posttransfection, *Gaussia* luciferase activity was measured in triplicates using diluted *Gaussia* substrate (1:12) and a microplate luminometer.

### Prediction of MFE of the RNA secondary structure.

Secondary structures of the first 1,000 nucleotides of the HIV-1 *env* gene were predicted using RNAfold (48) based on a loop-based energy model and a dynamic programming algorithm (49). Minimum free energies (MFEs) of the predicted structure were extracted.

### Generation of mean free energy-deviating mutant HIV-1 constructs.

To generate proviral constructs whose 5′ *env* regions have different predicted secondary structure mean free energies (MFEs), MFEs of a pool of random single point mutants that fulfilled the following criteria were calculated: (i) the predicted translated sequence of the *env* gene is not altered, (ii) no CpGs are introduced or removed, and (iii) no new AG or GU dinucleotides were introduced to avoid the creation of new splice donor or acceptor sites. MFEs of the mutants were compared, and single mutants that either increase or decrease the MFE of the LM-region structure were chosen. Single point mutations were introduced in the proviral constructs using the Q5 site-directed mutagenesis kit (New England Biolabs).

### Statistical analysis.

Statistical analysis was performed using GraphPad Prism software. Two-tailed unpaired Student’s *t* test, Mann-Whitney U-test, and one-sample *t* test (for comparison with normalized control) were used to determine statistical significance. Spearman’s nonparametric test was used for correlation analyses. Significant differences are indicated as follows: *, *P* < 0.05; **, *P* < 0.01; ***, *P* < 0.001; ****, *P* < 0.0001. Statistical parameters are specified in the figure legends.

### Data availability.

Nucleotide sequences used in the present study have the accession numbers shown in parentheses: SIVcol 1 (KF214241), SIVcol 2 (KF214240), SIVcol 3 (AF301156), SIVolc (FM165200), SIVwrc 1 (AM713177), SIVwrc 2 (AM745105), SIVlhoest (AF075269), SIVsun 1 (AF131870), SIVsun 2 (FR751162), SIVmon (AY340701), SIVgsn 1 (AF468658), SIVgsn 2 (AF468659), SIVmus 1 (AY340700), SIVmus 2 (EF070329), SIVsyk (AY523867), SIVcpz Pts (JQ768416), SIVgor 1 (KP004989), SIVgor 2 (KP004991), SIVcpz Ptt 1 (FR686510), SIVcpz Ptt 2 (JN835460), HIV-1 M C 1 (AF443075), HIV-1 M C 2 (AF067154), HIV-1 M C 3 (AF411966), HIV-1 M B3 (EF514701), HIV-1 M B4 (M19921), HIV-1 M B2 (JN944909), HIV-1 M E (AB032741), SIVrcm (HM803689), SIVmnd (AF367411), SIVdrl 1 (KM378564), SIVdrl 2 (KM378566), SIVagm sab (U04005), SIVagm 1 (AB253736), SIVagm tan (U58991), SIVsmm 2 (JX860423), SIVsmm 4 (JX860419), SIVsmm 5 (JX860417), SIVsmm 7 (JX860416), SIVmac239 (AY588946), SIVmac251 (KC522216), SIVsmm 1 (JX860413), SIVsmm 3 (JX860414), HIV-2 B EHO (U27200), HIV-2 1 B (KY025545), HIV-2 AB 1 (KX174311), HIV-2 AB 3 (AB499695), HIV-2 A 1 (M30895), HIV-2 A 2 (EU980602), HIV-2 A 4 (M15390), HIV-2 A 3 (D00835), HIV-2 A 6 (U22047), HIV-2 A 7 (KY025541), HIV-2 A 8 (M31113), feline immunodeficiency virus (FIV) (X57002.1), FIV (DQ192583.1), FIV (EF455611.1), FIV (MF352016.1), FIV (U11820.1), FIV (AY600517.1), FIV (AF474246.1), equine infectious anemia virus (EIAV) (JX480632.1), EIAV (JX480634.1), EIAV (JX480633.1), bovine immunodeficiency-like virus (BIV) (M32690.1), BIV (L04974), Moloney murine leukemia virus (MLV) (NC_001501.1), xenotropic MLV (JF908816.1), and Friend MLV (LC229035.1).
