# Supplementary material for: CpG Frequency in the 5′ Third of the env Gene Determines Sensitivity of Primary HIV-1 Strains to the Zinc-Finger Antiviral Protein
Source: mBio. 2020 Jan 14;11(1):e02903-19. doi: 10.1128/mBio.02903-19 (PMC6960287; doi:10.1128/mBio.02903-19)
Supplement: TABLE S1 [file mBio.02903-19-st001.docx]

**Table S1.** CpG frequencies in primate lentiviral genomes

| Name | Accession number | No of CpGs *gag* to *nef* | Length *gag* to  *nef* (bp) | No of CpGs *env* | Length of *env* (bp) | % CpG  *gag* to *nef* | % CpG *env* | No of GpCs *gag* to *nef* | % GpC  *gag* to *nef* |
| --- | --- | --- | --- | --- | --- | --- | --- | --- | --- |
| SIVcol BWC07 | KF214241 | 59 | 8032 | 27 | 2505 | 0.730 | 1.078 | 381 | 4.744 |
| SIVcol BWC01 | KF214240 | 64 | 8065 | 37 | 2529 | 0.790 | 1.463 | 384 | 4.761 |
| SIVcol CGU1 | AF301156 | 78 | 8002 | 34 | 2514 | 0.975 | 1.352 | 392 | 4.899 |
| SIVolc 12CI97 | FM165200 | 51 | 7973 | 21 | 2532 | 0.640 | 0.829 | 329 | 4.126 |
| SIVwrc 98CI04 | AM713177 | 38 | 8411 | 14 | 2655 | 0.452 | 0.527 | 349 | 4.149 |
| SIVwrc 97CI14 | AM745105 | 37 | 8372 | 18 | 2637 | 0.442 | 0.683 | 346 | 4.133 |
| SIVlst | AF075269 | 42 | 8447 | 20 | 2739 | 0.497 | 0.730 | 358 | 4.238 |
| SIVsun λ20L14/S2 | AF131870 | 46 | 8525 | 25 | 2760 | 0.540 | 0.906 | 397 | 4.657 |
| SIVsun K08_Gab | FR751162 | 39 | 8511 | 24 | 2790 | 0.458 | 0.860 | 402 | 4.723 |
| SIVmon 99CMCML1 | AY340701 | 206 | 8855 | 62 | 2664 | 2.326 | 2.327 | 457 | 5.161 |
| SIVgsn 99CM71 | AF468658 | 156 | 8804 | 62 | 2661 | 1.772 | 2.330 | 458 | 5.202 |
| SIVgsn 99CM166 | AF468659 | 157 | 8813 | 61 | 2646 | 1.781 | 2.305 | 466 | 5.288 |
| SIVmus 01CM1085 | AY340700 | 132 | 8821 | 56 | 2694 | 1.496 | 2.079 | 460 | 5.215 |
| SIVmus 01CM1246 | EF070329 | 164 | 8815 | 65 | 2649 | 1.860 | 2.454 | 453 | 5.139 |
| SIVsyk KE51 | AY523867 | 98 | 8511 | 44 | 2523 | 1.151 | 1.744 | 396 | 4.653 |
| SIVcpz *Pts* TAN13 | JQ768416 | 70 | 8730 | 27 | 2613 | 0.802 | 1.033 | 393 | 4.502 |
| SIVgor BPID1 | KP004989 | 53 | 8732 | 24 | 2670 | 0.607 | 0.899 | 404 | 4.627 |
| SIVgor BQID2 | KP004991 | 61 | 8744 | 28 | 2655 | 0.698 | 1.055 | 396 | 4.529 |
| SIVcpz *Ptt* 04Cam155 | FR686510 | 58 | 8722 | 25 | 2613 | 0.665 | 0.957 | 399 | 4.575 |
| SIVcpz *Ptt* EK505.c2 | JN835460 | 57 | 8596 | 26 | 2508 | 0.663 | 1.037 | 356 | 4.142 |
| HIV-1 M C 96BWMO3.2 | AF443075 | 59 | 8659 | 33 | 2595 | 0.681 | 1.272 | 396 | 4.573 |
| HIV-1 M C 301999 | AF067154 | 55 | 8681 | 23 | 2622 | 0.634 | 0.877 | 390 | 4.493 |
| HIV-1 M C 99ZASW7 | AF411966 | 60 | 8648 | 29 | 2547 | 0.694 | 1.139 | 370 | 4.278 |
| HIV-1 M B PMVL 049 | EF514701 | 60 | 8655 | 31 | 2595 | 0.693 | 1.195 | 389 | 4.495 |
| HIV-1 M B NL4-3 | M19921 | 63 | 8618 | 26 | 2565 | 0.731 | 1.014 | 376 | 4.363 |
| HIV-1 M B CH77_TF1 | JN944909 | 70 | 8652 | 30 | 2544 | 0.809 | 1.179 | 387 | 4.473 |
| HIV-1 M E 95TNIH047 | AB032741 | 59 | 8625 | 24 | 2586 | 0.684 | 0.928 | 365 | 4.232 |
| SIVrcm 02CM8081 | HM803689 | 75 | 8861 | 33 | 2586 | 0.846 | 1.276 | 391 | 4.413 |
| SIVmnd-2 | AF367411 | 92 | 8543 | 24 | 2601 | 1.077 | 0.923 | 375 | 4.390 |
| SIVdrl D4 | KM378564 | 89 | 8853 | 33 | 2652 | 1.005 | 1.244 | 408 | 4.609 |
| SIVdrl D6 | KM378566 | 83 | 8906 | 27 | 2691 | 0.932 | 1.003 | 398 | 4.469 |
| SIVsab agm.sab-1 | U04005 | 87 | 8585 | 26 | 2199 | 1.013 | 1.182 | 424 | 4.939 |
| SIVagm TYO-1 | AB253736 | 117 | 8460 | 50 | 2595 | 1.383 | 1.927 | 374 | 4.421 |
| SIVtan agm.tan-1 | U58991 | 124 | 8507 | 47 | 2604 | 1.458 | 1.805 | 406 | 4.773 |
| SIVsmm M940 | JX860423 | 97 | 8876 | 42 | 2700 | 1.093 | 1.556 | 417 | 4.698 |
| SIVsmm M923 | JX860419 | 93 | 8831 | 44 | 2673 | 1.053 | 1.646 | 390 | 4.416 |
| SIVsmm M919 | JX860417 | 91 | 8844 | 39 | 2682 | 1.029 | 1.454 | 388 | 4.387 |
| SIVsmm G932 | JX860416 | 92 | 8768 | 41 | 2661 | 1.049 | 1.541 | 382 | 4.357 |
| SIVmac 239 | AY588946 | 85 | 8816 | 39 | 2640 | 0.964 | 1.477 | 379 | 4.299 |
| SIVmac 251 | KC522216 | 93 | 8917 | 41 | 2664 | 1.043 | 1.539 | 385 | 4.318 |
| SIVsmm D215 | JX860413 | 93 | 8816 | 37 | 2655 | 1.055 | 1.394 | 408 | 4.628 |
| SIVsmm FTq | JX860414 | 98 | 8824 | 48 | 2646 | 1.111 | 1.814 | 400 | 4.533 |
| HIV-2 B EHO | U27200 | 115 | 8698 | 47 | 2559 | 1.322 | 1.837 | 391 | 4.495 |
| HIV-2 1 B LA44 | KY025545 | 121 | 8761 | 55 | 2616 | 1.381 | 2.102 | 420 | 4.794 |
| HIV-2 AB 7312A | KX174311 | 121 | 8725 | 55 | 2574 | 1.387 | 2.137 | 423 | 4.848 |
| HIV-2 AB 08JP.NMC842 | AB499695 | 107 | 8698 | 48 | 2547 | 1.231 | 1.885 | 410 | 4.714 |
| HIV-2 A GH-1 | M30895 | 147 | 8764 | 59 | 2559 | 1.677 | 2.306 | 412 | 4.701 |
| HIV-2 A NNVA | EU980602 | 147 | 8777 | 60 | 2574 | 1.675 | 2.331 | 425 | 4.842 |
| HIV-2 A ROD | M15390 | 118 | 8782 | 54 | 2577 | 1.344 | 2.096 | 426 | 4.851 |
| HIV-2 A CAM 2 | D00835 | 110 | 8794 | 50 | 2580 | 1.251 | 1.938 | 431 | 4.901 |
| HIV-2 A KR | U22047 | 128 | 8767 | 53 | 2574 | 1.460 | 2.059 | 432 | 4.928 |
| HIV-2 A LA40 | KY025541 | 143 | 8771 | 54 | 2571 | 1.630 | 2.100 | 423 | 4.823 |
| HIV-2 A ST | M31113 | 139 | 8782 | 54 | 2580 | 1.583 | 2.093 | 430 | 4.896 |
