# Supplementary figures and images for: CpG Frequency in the 5′ Third of the env Gene Determines Sensitivity of Primary HIV-1 Strains to the Zinc-Finger Antiviral Protein
Source: mBio. 2020 Jan 14;11(1):e02903-19. doi: 10.1128/mBio.02903-19 (PMC6960287; doi:10.1128/mBio.02903-19)

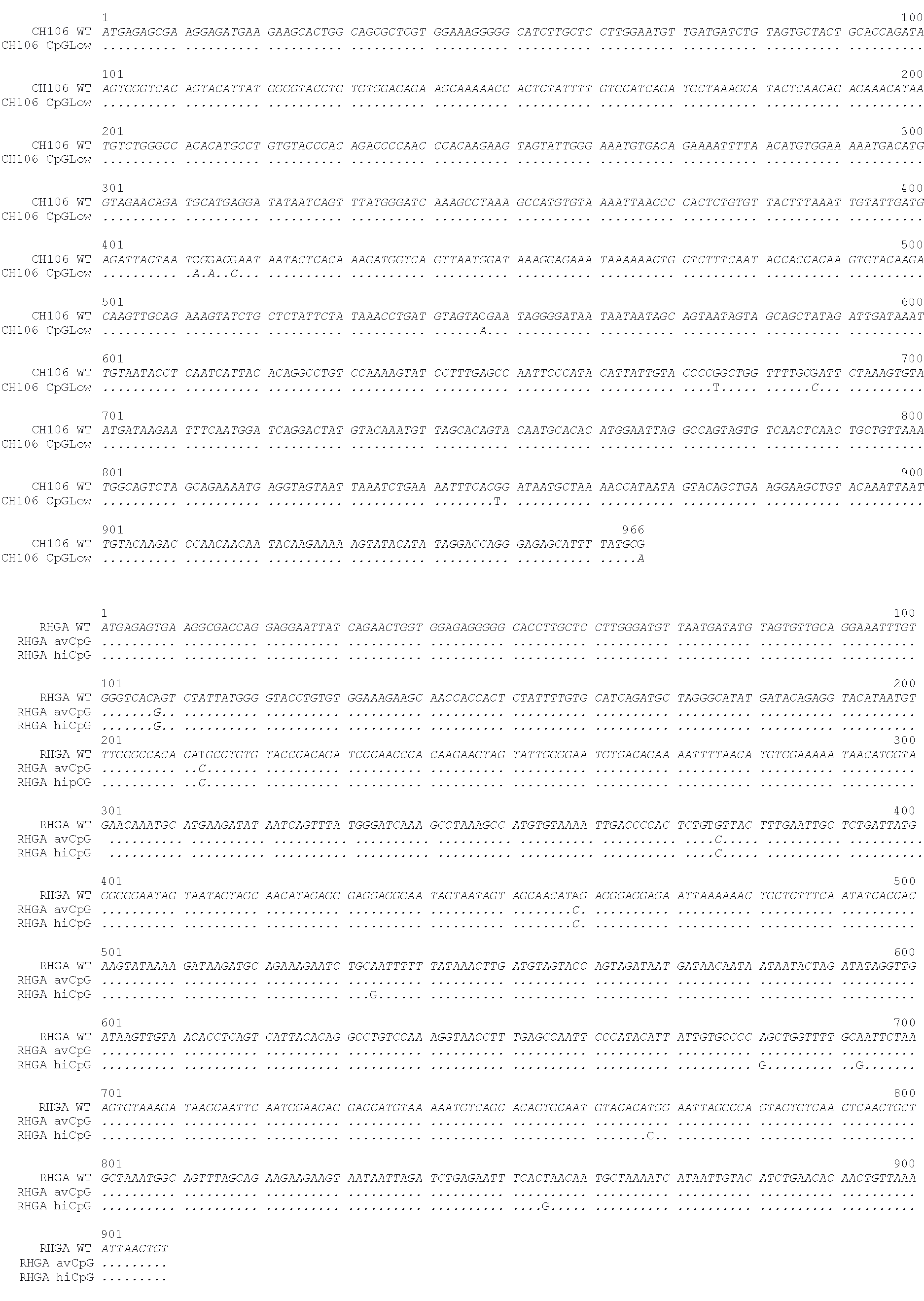

Supplement: FIG S5 [file mBio.02903-19-sf005.tif]
